# A projection from the paraventricular nucleus of the thalamus to the shell of the nucleus accumbens contributes to footshock stress-induced social avoidance

**DOI:** 10.1016/j.ynstr.2020.100266

**Published:** 2020-10-31

**Authors:** Xinwen Dong, Sa Li, Gilbert J. Kirouac

**Affiliations:** a Department of Oral Biology, Dr. Gerald Niznick College of Dentistry, Rady Faculty of Health Sciences, University of Manitoba, Winnipeg, Manitoba, R3E 0W2, Canada; b Department of Psychiatry, Max Rady College of Medicine, Rady Faculty of Health Sciences, University of Manitoba, Winnipeg, Manitoba, R3E 0W2, Canada; c Key Laboratory of Mental Health, Institute of Psychology, Chinese Academy of Sciences, Beijing, 100101, China

**Keywords:** Social anxiety, Paraventricular nucleus of the thalamus, Nucleus accumbens, Dynorphin, Social avoidance

## Abstract

The paraventricular nucleus of the thalamus (PVT) is an area of the dorsal midline thalamus that contributes to footshock induced anxiety. The PVT sends a dense projection to the shell of the nucleus accumbens (NAcSh) and the present study explored if this projection is involved in the behavioral changes produced by a single exposure of rats to inescapable footshocks. The inhibitory Designer Receptors Exclusively Activated by Designer Drugs (DREADDs) hM4Di was transduced in PVT neurons that project to the NAcSh. Rats were exposed to an episode of moderately intense footshock (1.5 mA × 2 s × 5) and assigned to either high-responder (HR) or low-responder groups (LR) according to their level of fear generalization 24 h later. The effect of chemogenetic inhibition of the PVT-NAcSh projection on anxiety- and fear-like behaviors was assessed at approximately 2 weeks post-footshock. HR showed a higher level of social avoidance compared to non-shocked animals and LR. The elevated level of social avoidance was attenuated in the HR treated with the hM4Di agonist clozapine (0.01 mg/kg, i.p.) or clozapine N-oxide (CNO) administrations in the NAcSh while avoidance of open spaces and contextual fear expression were not affected. Analysis of protein product of the early to immediate gene *cfos* indicated that these effects were mediated by dynorphin neurons in the NAcSh. This study provides evidence for a role of a projection from the PVT to the NAcSh in stress-induced social avoidance independent of anxiety to non-social stimuli and contextual fear mechanisms.

## 1 Introduction

An association between stress and anxiety is well established with stress being a contributing factor for most anxiety disorders including posttraumatic stress disorder (PTSD) and social anxiety disorder (Shin and Liberzon, 2010; Bystritsky and Kronemyer, 2014; Daviu et al., 2019). It is generally accepted that PTSD can be triggered when susceptible individuals are exposed to an intense fear-inducing and/or stressful experience (Collimore et al., 2010; McMillan et al., 2017). The enduring effect of an acute and intense stress experience on behavior is not unique to humans seeing that exposure of rodents to a single episode of footshocks produces anxiety including a heightened level of social avoidance (Louvart et al., 2005; Siegmund and Wotjak, 2007; Mikics et al., 2008; Chen et al., 2012) for up to four weeks post-shock in susceptible rats (Chen et al., 2012).

Pharmacological evidence indicates that the paraventricular nucleus of the thalamus (PVT) located in the dorsal midline thalamus contributes to fear and anxiety in rats that are exposed to a single episode of inescapable footshocks (Li et al., 2010a; Dong et al., 2015). The PVT is one of the areas of the brain most consistently reported to be activated by aversive conditions (Hsu et al., 2014; Kirouac, 2015) and anatomical studies have established that the PVT receives afferent input from a number of brain regions known to be activated by stress (Otake et al., 2002; Li and Kirouac, 2012; Kirouac, 2015). The PVT projects densely to subcortical areas implicated in defensive behaviors including the shell of the nucleus accumbens (NAcSh), dorsolateral region of the bed nucleus of the stria terminalis (BSTDL) and the lateral region of the central nucleus of the amygdala (CeL) (Parsons et al., 2007; Li and Kirouac, 2008; Vertes and Hoover, 2008), indicating that the PVT is well-positioned to regulate the behavioral responses to stress. For instance, the PVT has been shown to contribute to behavioral freezing to conditioned fear cues (Padilla-Coreano et al., 2012; Li et al., 2014; Do-Monte et al., 2015; Kirouac, 2015; Penzo et al., 2015) via a projection to the CeL (Do-Monte et al., 2015; Penzo et al., 2015). However, the projection by which the PVT influences the social avoidance after exposure to footshock-stress has not been identified. The NAcSh is emerging as an area of the striatum critical for social interaction where disruption of normal signaling contributes to social avoidance (Aragona et al., 2006; Christoffel et al., 2011; Chaudhury et al., 2013; Dolen et al., 2013; Gunaydin et al., 2014; Francis et al., 2015; Resendez et al., 2016; Wook Koo et al., 2016; Rademacher et al., 2017; Folkes et al., 2019; Steinman et al., 2019). A detailed quantitative analysis of the number of neurons in the PVT that project to the NAcSh and other subcortical areas demonstrated that most of the neurons in the PVT project to the NAcSh (Dong et al., 2017). Here we investigated if PVT neurons that project to the NAcSh contribute to the stress-induced behavioral changes observed after exposure of rats to a single episode of inescapable footshocks. We used a chemogenetic approach to inhibit PVT neurons that project to the NAcSh or PVT fibers in the NAcSh to show that a PVT projection to the NAcSh contributes to social avoidance in stress-susceptible rats.

## 2 Materials and methods

### 2.1 Subjects

A total of 120 adult male Sprague-Dawley rats (University of Manitoba vivarium) were used for the experiment. Animals were housed in cages kept in a climate controlled room maintained on a 12 h/12 h light/dark cycle (lights on at 07:00). All rats had free access to food and water through the experiment and were handled for 2 min on alternate days during a 7-day adaptation period. All the behavioral procedures were done in the light cycle of the day (09:00–17:00) and were according to guidelines of the Canadian Council on Animal Care and approved by Research Ethics Review Board of the University of Manitoba.

### 2.2 General experimental procedures

**Fig. 1:**
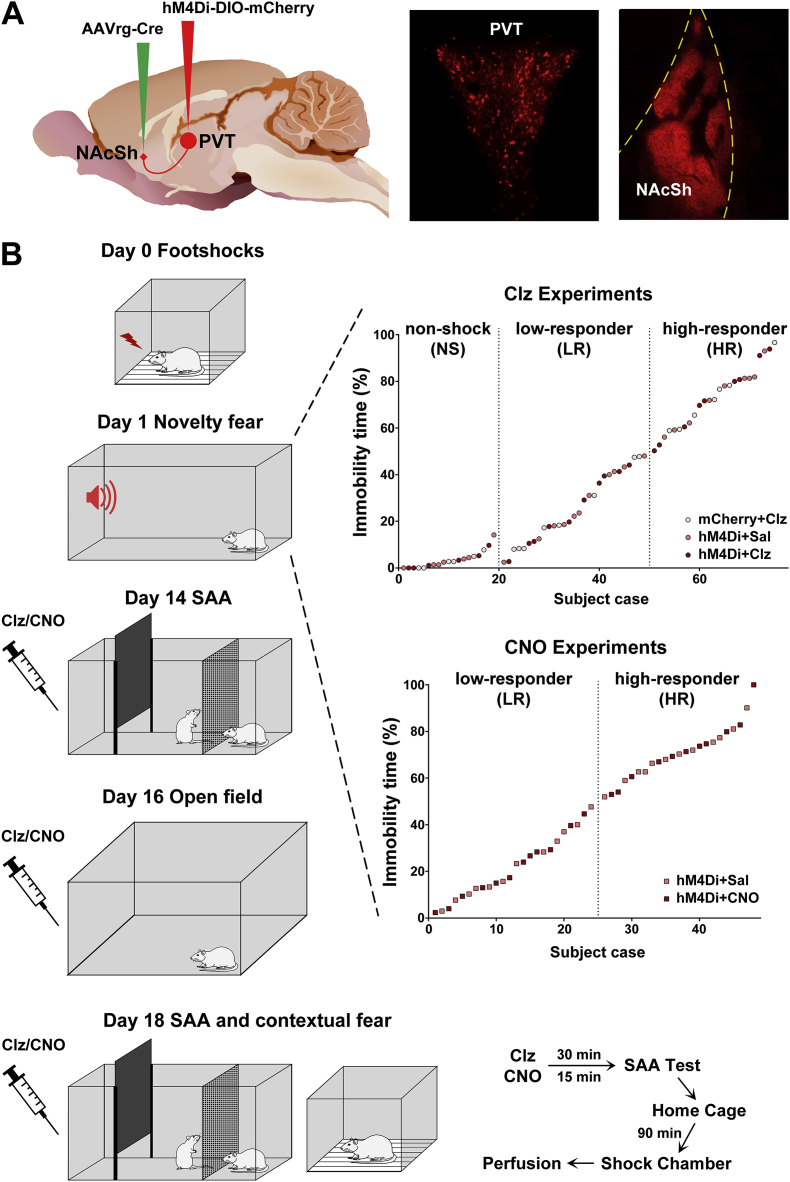
Viral injections and behavioral procedures. (A) Diagram of the intersectional approach to transduce hM4Di in the PVT neurons (left side) and examples of mCherry expression in PVT neurons and fibers in the NAcSh (right side). (B) Sequence of the behavioral procedures (left side) and assignment of shocked rats into the LR and HR groups based on the immobility to a novel environment 24 h after footshock exposure (right side). The sagittal brain image in (A) was created by Gill Brown, King's College, London. (For interpretation of the references to color in this figure legend, the reader is referred to the Web version of this article.)

After adaptation to handling, viral vectors were microinjected into the NAcSh and the PVT to transduce the expression of the inhibitory designer receptor hM4Di in PVT neurons and fibers that innervate the NAcSh (Fig. 1A). The effects of hM4Di activation (i.e., chemogenetic inhibition of PVT neurons that innervate the NAcSh) using systemic injections of clozapine as an agonist (Gomez et al., 2017) were examined in tests of fear and anxiety. Some neurons in the PVT that innervate the NAcSh provide collateral innervation of the BSTDL and CeL (Dong et al., 2017). Therefore in another group of subjects, clozapine N-oxide (CNO) was administered directly in the NAcSh via implanted cannulas to activate hM4Di on PVT fibers in the NAcSh to provide further evidence that the effects of systemic injections of clozapine were mediated by PVT fibers in the NAcSh. The behavioral procedures were started seven days after the viral injections (Fig. 1B). Rats were given inescapable footshocks (Day 0) and based on the amount of immobility displayed by shocked rats placed in a novel chamber (Day 1), subjects were assigned to the high-responder (HR) or low-responder (LR) groups as previously done (Chen et al., 2012). The effect of activating hM4Di in a social approach-avoidance (SAA) test was assessed 14 days after footshock exposure (Day 14) and behavioral activity in an open field was assessed on Day 16. The effect of activation of hM4Di in the SAA test was re-examined in the same rats with the drug treatment counterbalanced on Day 18 (rats treated with clozapine or CNO on Day 14 were treated with saline on Day 18). After the SAA test on Day 18, rats were placed in their home cages for 90 min before being transferred to the shock chamber for determining the effect of chemogenetic inhibition of the PVT-NAcSh projection on contextual fear expression. Immediately after the contextual fear expression test, rats were anesthetized and perfused with fixative for subsequent histological analysis of the reporter gene mCherry, cannula placement and the expression of cFos in the PVT, NAcSh, BSTDL and CeL. It is important to understand that the expression of cFos peaks at approximately 90 min after exposure to an activating stimulus (Dragunow and Faull, 1989). Since the rats were given an anesthetic and perfused after the exposure to the shock context and 90 min after the SAA test, the cFos expression levels reported in this paper are related to the SAA test and not the contextual fear test. Accordingly, neuronal expression of cFos in the PVT and the NAcSh was used as a readout that the chemogentic manipulations using systemic injections of clozapine did indeed inhibit the PVT-NAcSh projection.

### 2.3 Surgical procedure and viral injections

Animals were anesthetized with 2–3% isoflurane and given meloxicam (2 mg/kg, s.c.) for post-surgery pain management. Animals were placed in a Stoelting stereotaxic frame and a hand drill was used to expose the brain surface above the target sites. Viral vectors were injected at a rate of 80 nl/min using glass pipettes (outer diameter of approximately 40 μm) connected to a pressure injection device (Picospritzer, Park Hannifin, Hollis, NH, USA). Injections of 350 nl of AAV2-retro-Syn1-EBFP-Cre (2.9 × 10^13^ vg/ml, #51507-AAVrg, AddGene, Cambridge, MA, USA) were done bilaterally in the medial region of the NAcSh (1.5 mm anterior and 0.9 mm lateral to bregma; and 7.2 mm ventral to the dorsal surface of the bone). Following this, 600 nl of AAV8-hSyn-DIO-hM4Di-mCherry (1.4 × 10^13^ vg/ml, #44362-AAV8, AddGene) or AAV8-hSyn-DIO-mCherry (1.6 × 10^13^ vg/ml, #50459-AAV8, AddGene) was injected in the anterior PVT (aPVT) and posterior PVT (pPVT) to transduce neurons in both the aPVT and pPVT. Viral injections in the PVT were done with the pipette holder angled at a 10° from the midline on the right side of the sagittal sinus according to the following coordinates: aPVT (1.8 mm posterior and 1.05 mm lateral to bregma; and 5.9 mm ventral to the surface of the bone) and pPVT (3.1 mm posterior and 1.0 mm lateral to bregma; and 5.8 mm ventral to the surface of the bone). The pipette was kept in the brain for 10 min following the injections to prevent leakage and contamination along the pipette track. Subgroups of rats were implanted with bilateral stainless steel guide cannula (23 gauge, RWD Life Science, Shenzhen, China) directed at the NAcSh (1.5 mm anterior and 0.9 mm lateral to bregma; and 5.2 mm ventral to the dorsal surface of the bone). Stainless steel screws and dental cement were used to secure the guide cannula in place and capped with a stylet. The scalp incision was sutured and rats were returned to their home cages for recovery when fully awake. The use of this intersectional viral strategy results in the expression of the inhibitory receptor, hM4Di in a Cre-dependent manner in PVT neurons that project to the NAcSh (Fig. 1A).

### 2.4 Footshock procedure and grouping of shocked rats

Seven days after injections of viruses, rats were transferred one at a time to a room dedicated for the delivery of footshocks. After a 2 min acclimation period, each rat received 5 footshocks (1.5 mA, 2 s, randomly presented at 10–50 s intervals over 2 min) through the grid floor of a shock chamber (MED Associates, St. Albans, Vermont, USA). The rats were kept in the chamber for another 60 s before being returned to their home cages. Nonshocked (NS) rats were placed in the shock chamber for the same amount of time. The shock chamber was cleaned with alcohol (10%) and new bedding under the grid floor was used for each rat. Twenty-four hours later, rats were placed in a rectangular open field (65 × 40 × 50 cm; illuminated at 3–5 lx) for 6 min with the presence of a novel tone (9 kHz, 75 dB) during the last 3 min. Behavioral immobility defined as no movement of four limbs was quantified from the recorded video by experimenters blind to the conditions. The percentage of the total immobility time during the 6-min test was used to assign shocked rats into HR and LR groups using a cut-off criterion of 50% of immobility time as this cut-off effectively produces groups with low and high levels of anxiety lasting for up to 4 weeks post-shock (Chen et al., 2012).

### 2.5 Social approach-avoidance (SAA) test

Social avoidance was examined in a weakly illuminated room (3–5 lx) using a three-compartment black Plexiglas chamber consisting of a holding compartment (20 × 40 × 50 cm) with a sliding door facing an interaction compartment (30 × 40 × 50 cm) adjacent to another compartment with a mesh wall (15 × 40 × 50 cm). After a 3 min habituation period, the sliding door was opened to allow the test rat to interact with a non-familiar male rat located in the meshed compartment for up to 10 min. The behavior was recorded by a video camera mounted on the ceiling and analyzed manually for the amount of time spent in the region of the interaction compartment immediately adjacent to the unfamiliar rat (social zone, 15 × 40 × 50 cm). The behavior of the test rat was quantified from the video by two experimenters blind to the conditions.

### 2.6 Open field test

The open field test was done in a square open field (80 × 80 × 40 cm) made of black Plexiglas which was illuminated at 3–5 lx. The rat was placed into one corner of the open field and allowed to freely explore the open field for 5 min. The behavior was recorded by a video tracking system Ethovision (Noldus, Wageningen, Netherland) and the total amount of time spent in the center of the open field (35 × 35 cm) and distance moved were quantified. The amount of time of the rat spent immobile was also quantified visually by two experimenters blind to the conditions.

### *2.7* *Shock context re-*exposure

Rats were placed into the shock chamber for 5 min and the total time the rat spent freezing was quantified by experimenters blind to the conditions.

### 2.8 Drug administration

Clozapine (Tocris, Minneapolis, MN) was dissolved in distilled water (pH = 4) as 3 mM stock solution. On the test day, the stock solution was dissolved with 0.9% sterile saline into 30 μM solution and administered intraperitoneally (i.p.) at a dose of 0.01 mg/kg. Clozapine or saline injection were given 30 min before behavioral tests and the rat returned to its home cage before being placed in one of the test situations. CNO (RTI International, NC, USA) was dissolved in 0.9% sterile saline as 3 mM stock solution. On the test day, the stock solution was dissolved in 0.9% sterile saline as a 3 μM solution. The rats received 0.5 μl of CNO or vehicle bilaterally in the NAcSh through a double-injector cannula (27 gauge, RWD Life Science) which protruded 2.0 mm below the guide cannula. Infusions were delivered at the rate of 1 μl/min over 0.5 min with an infusion pump (Genie, Kent Scientific, CT, USA) and 1 ml syringe connected to the injector cannula with polyethylene tubing. The injectors were kept in the guide cannula for 5 min following the injections to prevent leakage. The stylet was inserted back into the guide cannula and the rat was returned to its home cage for 15 min before a behavioral assessment. Mock microinjection involving the removal and the reinsertion of the stylet were done in the drug delivery room for 3 days prior to the experiment to habituate the animals to the handling and injection procedure.

### 2.9 Immunoreactions

Rats were deeply anesthetized with 10% chloral hydrate (600 mg/kg, i.p.) and perfused transcardially with 200 ml heparinized saline followed by 500 ml ice-cold 4% paraformaldehyde in 0.1 M PB (pH 7.4). The brains were removed and post-fixed in the same fixative for 24 h followed by cryoprotection in 20% sucrose with 10% glycerin over 2 days at 4 °C. Coronal sections of brain regions that included the NAcSh and the PVT were taken at 50 μm using a cryostat (Vibratome Ultrapro 5000). Brain sections were pre-incubated in a blocking solution containing 5% donkey serum, 0.3% Triton X-100 and 0.1% sodium azide in PBS for 1 h and then incubated in primary rabbit anti-cFos antibody (1:2000; ABE457, Millipore, Temecula, CA, USA) and mouse anti-mCherry antibody (1:3000; Takara Bio, Mountain View, CA, USA) overnight. After 3 rinses in PBS, sections were transferred to secondary donkey anti-rabbit antibody conjugated to Alexa Fluor 488 (1:1000; A21206, Invitrogen, Calsbad, CA, USA) and donkey anti-mouse antibody conjugated to Cy 3 (1:500; 715-165-151, Jackson ImmunoResearch, West Grove, PA, USA) for 2 h. Sections of the NAcSh were reacted for preproenkephalin, the precursor peptide for enkephalin and preprodynorphin, the precursor peptide for dynorphin, in addition to cFos to assess if medium spiny neurons synthesizing these opioid neuropeptides were activated in rats exposed to the SAA test. Sections of the NAcSh were incubated in primary antibodies against ENK (rabbit anti-preproenkephalin; 1:1000; E-3220-24, USBiological, Salem, MA, USA) and DYN (guinea pig anti-preprodynorphin; 1:2000; GP10110, Neuromics, Edina, MN, USA) in addition to mouse anti-cFos primary antibody (1:500; ab208942, Abcam, Cambridge, UK) overnight. After 3 rinses in PBS, sections were transferred to secondary antibodies containing donkey anti-rabbit antibodies conjugated to Alexa Fluor 647 (1:1000; A31573, Invitrogen), donkey anti-guinea pig antibodies conjugated to Cy3 (1:500; 706-165-148, Jackson ImmunoResearch) and donkey anti-mouse antibodies conjugated to Alexa Fluor Plus 488 (1:3000; A32766, Invitrogen) for 2 h. After three final rinses, sections were mounted, and coverslipped with fluorescent protectant mounting medium Fluoromount-G (SouthernBiotech, Birmingham, AL, USA) for subsequent examination.

### 2.10 Image analysis

Brain sections were examined and photographed using a Zeiss Axio Observer Z1 microscope equipped with Axiocam 503 mono camera. Images were taken under 20X magnification, numbers of X and Y stacks were set to cover the area of interests while Z-stacks were set to cover the full depth of visible cells with a depth interval of 0.56 μm. The exposure time was adjusted for each individual channel to optimize the images captured and was set at a consistent level for the different cases. The captured images were processed with Zen Blue Software (Zeiss) using the “Stitch” application for infusing the X and Y stacks and the “Extended depth of focus” application for the Z-stacks to obtain the maximum depth of field. The images shown are stacks compiled and with the contrast adjusted in Photoshop.

The number of single and double-labeled neurons for mCherry, enkephalin, dynorphin and cFos were quantified using ZEN Blue software (Zeiss) by experimenters blind to the conditions. All brain images were counted under the same gamma setting for each channel. The labeled neurons were manually marked by examining merged color channels of the images by verifying the presence of individual markers by alternating through the different color channels when the merge signal was ambiguous. The number of marked neurons was then calculated per mm^2^ area. For cFos and mCherry cell counts in the PVT, 12 sections representing the various anterior to posterior aspects of the PVT were quantified. For cFos, enkephalin, and dynorphin cell counts in the NAcSh, an anterior (+2.28 mm relative to bregma), middle (+1.80 mm relative to bregma) and posterior level (+1.44 mm relative to bregma) of the NAcSh was quantified, which included the areas of the NAcSh innervated by the PVT where mCherry fibers were present. The number of cFos positive nuclei were also counted from sections of the BSTDL (3 levels ranging from +0.12 mm to - 0.24 mm relative to bregma) and CeL (3 levels raning from - 2.28 mm to - 2.92 mm relative to bregma). The number shown in the figures and analyzed represents the number of neurons per mm^2^ counted bilaterally in the NAcSh, BSTDL, and CeL from the same subjects that had received the critical treatments and that were selected randomly.

### 2.11 Experimental design and statistical analyses

Behavioral data including immobility time, freezing time, time spent in social zone, and time spent in the center of the open field are presented as percentage of the total test period time. There were three chemogenetic conditions: 1) hM4Di and clozapine injection (chemogenetic inhibition); 2) hM4Di and saline injection (control for clozapine injection); 3) mCherry and clozapine injection (control for hM4Di). Control groups for mCherry alone and saline injection were not used since the major critical factors required to unambigously interpret the results were present. Two-way ANOVA was used for analyzing the effects of chemogenetic inhibition of PVT neurons and fibers that project to the NAcSh followed by *post-hoc* pairwise comparison and simple effect test with Sidak multiple comparisons tests. Statistical analyses were conducted in GraphPad Prism 8 (GraphPad Software, San Diego, CA, USA). A value of *p* < 0.05 was considered to be significant and the data were presented as mean ± SEM.

## 3 Results

### 3.1 Chemogenetic inhibition of the PVT projection to the NAcSh attenuates the social avoidance produced by footshocks

**Fig. 2:**
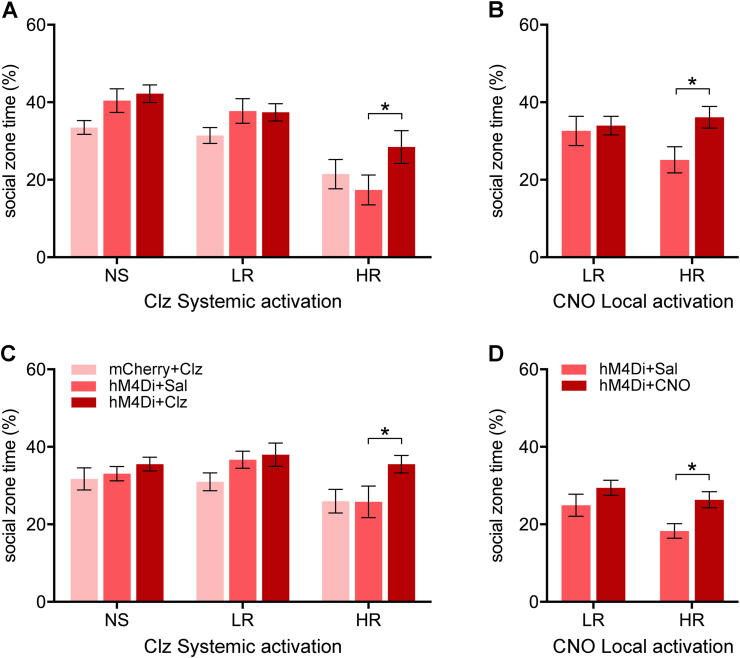
Effects of chemogenetic inhibition of the PVT-NAcSh projection on social approach-avoidance. Effects of chemogenetic inhibition of the PVT-NAcSh projection on SAA on Day 14 (A–B) and Day 18 (C–D) in rats treated with systemic injections of clozapine (Clz) to activate hM4Di (left side) or local administrations of CNO in the NAcSh (right side). Data are means ± SEM. *p < 0.05. Group sizes are *n* = 6 to 12. (NS + mCherry + Clz, *n* = 6; NS + hM4Di + Sal, *n* = 7; NS + hM4Di + Clz, *n* = 6; LR + mCherry + Clz, *n* = 8; LR + hM4Di + Sal, *n* = 11; LR + hM4Di + Clz, *n* = 10; HR + mCherry + Clz, *n* = 7; HR + hM4Di + Sal, *n* = 9; HR + hM4Di + Clz, *n* = 9; LR + hM4Di + Sal in NAcSh, *n* = 12; LR + hM4Di + CNO in NAcSh, *n* = 12; HR + hM4Di + Sal in NAcSh, *n* = 12; HR + hM4Di + CNO in NAcSh, *n* = 11.)

Experimental groups consisted of NS and shocked rats with hM4Di or mCherry where the shocked rats were further grouped into the anxiety susceptibility groups according to immobility to a novel environment 24 h after footshock exposure (i.e., HR and LR groups as shown in Fig. 1). The amount of time spent in the social zone was significantly different between the NS, LR and HR groups in the SAA test done on Day 14 (Fig. 2A, *F*_(2,64)_ = 20.1, *p* < 0.001) and Day 18 (Fig. 2C, *F*_(2,64)_ = 3.75, *p* = 0.029) with the HR group spending less time in the social zone (i.e., social avoidance) than the NS and LR groups on both test days (Fig. 2A, Day 14, HR vs NS: *p* < 0.001; HR vs LR: *p* < 0.001; Fig. 2C, Day 18, HR vs LR: *p* = 0.027).

Chemogenetic inhibition of PVT-NAcSh projecting neurons had a significant effect on the time spent in the social zone on Day 14 (Fig. 2A, *F*_(2,64)_ = 3.47, *p* = 0.037) with clozapine treated HR + hM4Di rats showing a significantly increased time in the social zone compared to the HR + hM4Di rats treated with saline (*p* = 0.037). In separate groups of shock rats with implanted cannulas, administrations of CNO in the NAcSh to inhibit PVT fibers locally in the NAcSh in HR + hM4Di rats resulted in an increase in social zone time on Day 14 compared to animals that received only saline (Fig. 2B, *p* = 0.037). On Day 18, chemogenetic inhibition of PVT-NAcSh projecting neurons had a significant effect on the time spent in the social zone (Fig. 2C, *F*_(2,64)_ = 4.13, *p* = 0.020) with clozapine treated HR + hM4Di rats showing significantly increased time in the social zone compared to the HR + hM4Di rats treated with saline (*p* = 0.039). Similar results were obtained from local inhibition of PVT fibers in the NAcSh in that HR + hM4Di rats received CNO administrations in the NAcSh displaying an increase in social zone time (Fig. 2D, *p* = 0.038) compared to rats treated with the vehicle. In summary, the results of the SAA test provide evidence that chemogenetic inhibition of PVT neurons that project to the NAcSh and local inhibition of PVT fibers in the NAcSh selectively reduce social avoidance in HR rats tested 14–18 days after footshock exposure. The fact that the effects of the clozapine and CNO treatment were reproduced on Day 18 using a counterbalanced drug design adds credibility of the findings.

### 3.2 Chemogenetic inhibition of the PVT projection to the NAcSh does not affect exploratory behavior in the open field or contextual fear expression

**Fig. 3:**
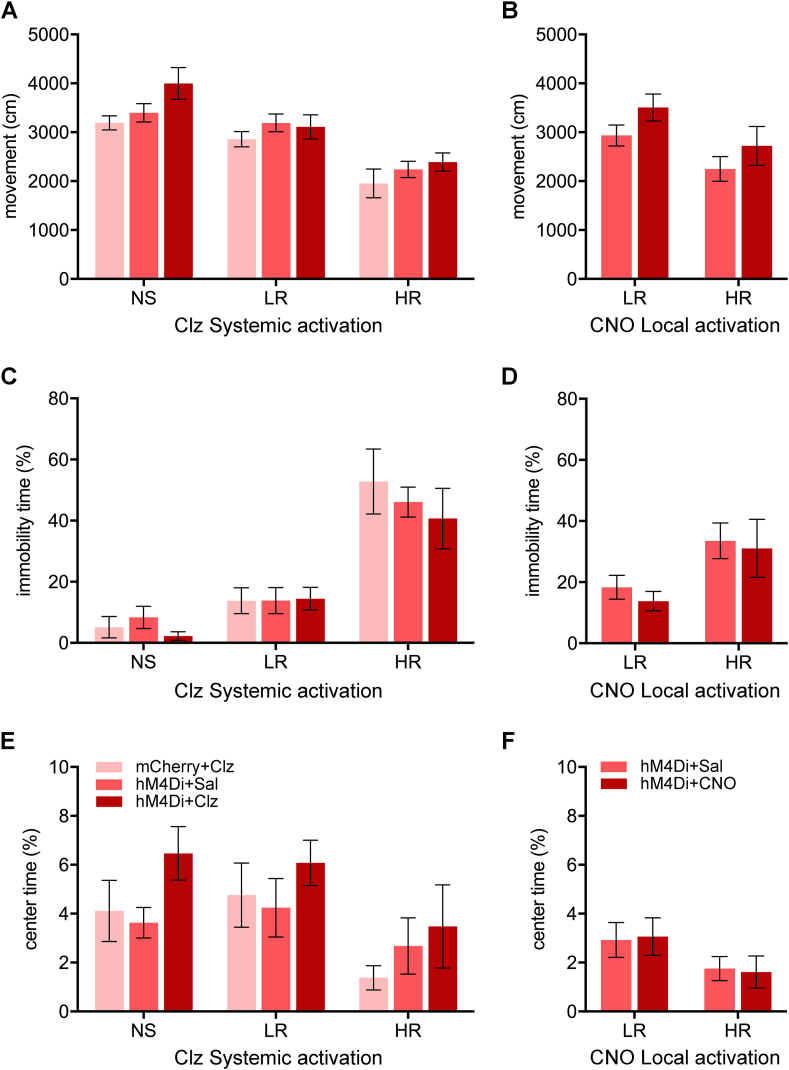
Effects of chemogenetic inhibition of the PVT-NAcSh projection on open field behaviors. Effects of chemogenetic inhibition of the PVT-NAcSh projection on locomotion (A–B), immobility (C–D), and time spent in the center (E–F) in rats treated with systemic injections of clozapine (Clz) to activate hM4Di (left side) or local administrations of CNO in the NAcSh (right side). Data are means ± SEM. Group sizes are *n* = 6 to 12. (NS + mCherry + Clz, *n* = 6; NS + hM4Di + Sal, *n* = 7; NS + hM4Di + Clz, *n* = 6; LR + mCherry + Clz, *n* = 8; LR + hM4Di + Sal, *n* = 11; LR + hM4Di + Clz, *n* = 10; HR + mCherry + Clz, *n* = 7; HR + hM4Di + Sal, *n* = 9; HR + hM4Di + Clz, *n* = 9; LR + hM4Di + Sal in NAcSh, *n* = 11; LR + hM4Di + CNO in NAcSh, *n* = 12; HR + hM4Di + Sal in NAcSh, *n* = 11; HR + hM4Di + CNO in NAcSh, *n* = 11.)

On Day 16, locomotion in the open field was reduced in the HR and LR groups (Fig. 3A, *F*_(2,64)_ = 27.51, *p* < 0.001) with the HR group having less locomotion than the NS and LR groups (HR vs NS: *p* < 0.001; HR vs LR: *p* < 0.001) and the LR group having less locomotion than the NS group (LR vs NS: *p* = 0.031). There was a significant difference in locomotion between the shock groups in the experiments involving systemic injections of clozapine (Fig. 3 **A**, *F*_(2,64)_ = 3.72, *p* = 0.030). There was no significant difference in locomotion between the HR + hM4Di group receiving CNO and saline in the NAcSh (Fig. 3B, *p* = 0.46) but a significant difference in locomotion between LR and HR (*F*_(1,41)_ = 6.27, *p* = 0.016).

The HR group displayed more immobility in the open field (Fig. 3C, *F*_(2,64)_ = 37.23, *p* < 0.001) than the NS and LR groups (HR vs NS: *p* < 0.001; HR vs LR: *p* < 0.001) whereas the LR group had similar immobility as the NS group (LR vs NS: *p* = 0.24). The chemogenetic conditions associated with systemic clozapine experiments did not have an effect on immobility (Fig. 3C, *F*_(2,64)_ = 0.49, p = 0.62) nor did injections of CNO in the NAcSh (Fig. 3D, *F*_(1,41)_ = 0.34, *p* = 0.56). Footshocks also had an effect on the time spent in the center of the open field (Fig. 3E, *F*_(2,64)_ = 4.06, *p* = 0.022) with the HR group spending less time in the center compared to the LR group (*p* = 0.028). The chemogenetic conditions had no effect on the time spent in the center (Fig. 3E, *F*_(2,64)_ = 2.38, *p* = 0.10). In addition, injections of CNO in the NAcSh did not produce detectable differences in the time spent in the center (Fig. 3F, *F*_(1,41)_ = 0.00, *p* = 0.99).

**Fig. 4:**
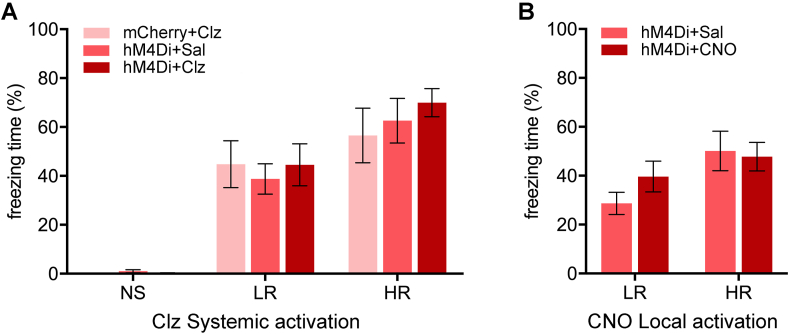
Effects of chemogenetic inhibition of the PVT-NAcSh projection on contextual fear. Effects of chemogenetic inhibition of the PVT-NAcSh projection on freezing to the shock context in rats treated with systemic injections of clozapine (Clz) to activate hM4Di (A) or local administrations of CNO in the NAcSh (B). Data are means ± SEM. Group sizes are *n* = 6 to 11. (NS + mCherry + Clz, *n* = 6; NS + hM4Di + Sal, *n* = 6; NS + hM4Di + Clz, *n* = 7; LR + mCherry + Clz, *n* = 8; LR + hM4Di + Sal, *n* = 10; LR + hM4Di + Clz, *n* = 11; HR + mCherry + Clz, *n* = 7; HR + hM4Di + Sal, *n* = 9; HR + hM4Di + Clz, *n* = 9; LR + hM4Di + Sal in NAcSh, *n* = 11; LR + hM4Di + CNO in NAcSh, *n* = 11; HR + hM4Di + Sal in NAcSh, *n* = 10; HR + hM4Di + CNO in NAcSh, *n* = 10.)

Contextual fear expression was examined on Day 18 (Fig. 4A, *F*_(2,64)_ = 45.12, *p* < 0.001) and both the LR and HR groups displayed more freezing than the NS groups (LR vs NS, *p* < 0.001; HR vs NS, *p* < 0.001) whereas the freezing level of the HR group was also higher than the LR group (*p* = 0.004). The chemogenetic inhibition using systemic clozapine as the hM4Di agonist did not affect freezing (Fig. 4A, *F*_(2,64)_ = 0.32, *p* = 0.73) nor did injections of CNO in the NAcSh (Fig. 4B, *F*_(1,38)_ = 1.02, *p* = 0.32).

### 3.3 Histological analysis related to the intersectional approach

**Fig. 5:**
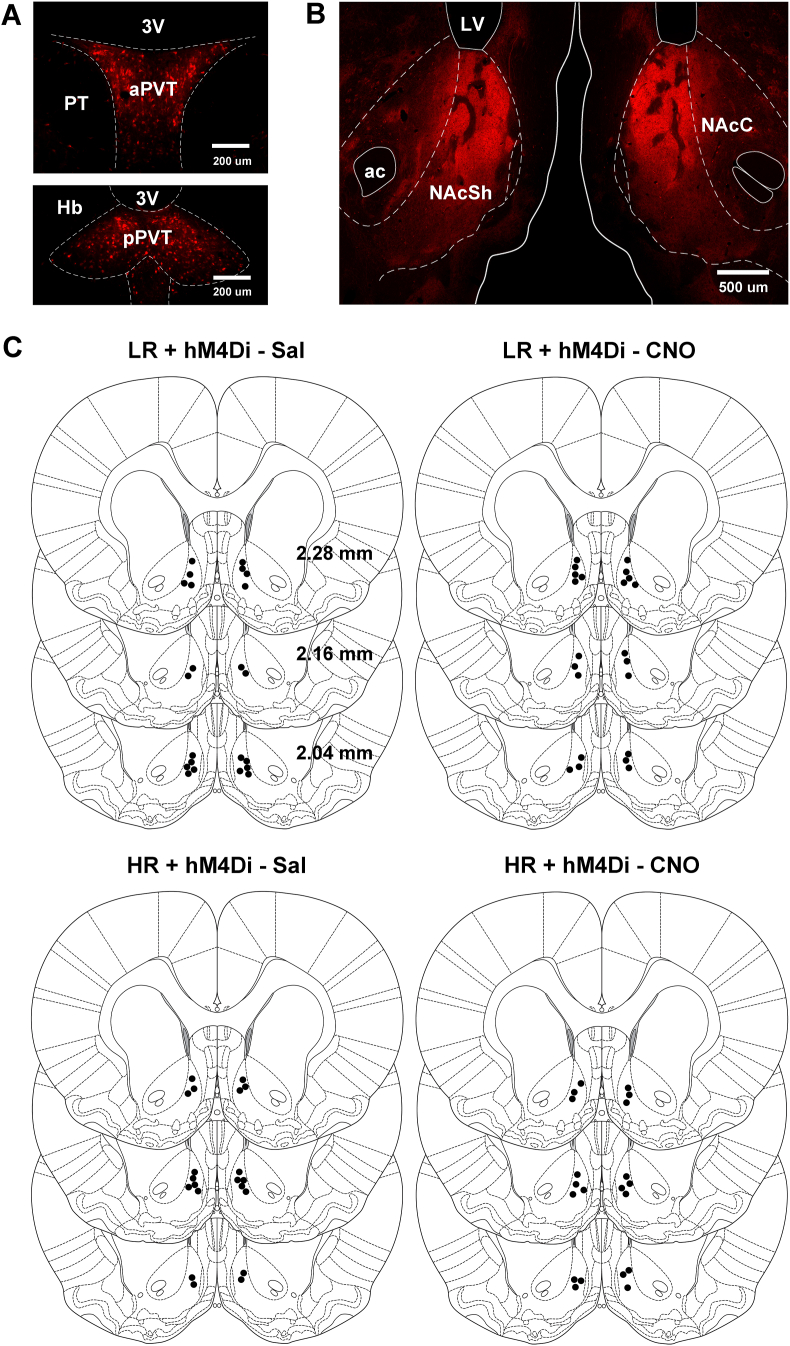
Immunofluorescence for hM4Di/mCherry and cannula placements. Representative examples of neurons in the aPVT and pPVT transduced with hM4Di/mCherry (A) and fibers in the NAcSh (B). (C) Locations of the injector cannula in the NAcSh. Scale bars: A, 200 μm; B, 500 μm. Abbreviations: 3 V, third ventricle; ac, anterior commissure; aPVT, anterior PVT; Hb, habenula; LV, lateral ventricle; NAcC, core of NAc; NAcSh, shell of NAc; pPVT, posterior PVT; PT, paratenial nucleus of the thalamus.

The intersectional viral strategy resulted in hM4Di/mCherry immunofluorescence in neurons located in the aPVT and pPVT (Fig. 5A). The mediodorsal and paratenial nuclei adjacent to the PVT were devoid of labeled neurons indicating that the intersectional strategy had high anatomical specificity for the PVT. The location of the transduced neurons in the PVT and fibers in the NAcSh observed with intersectional viral approach is consistent with previous studies using traditional retrograde and anterograde tracers (Parsons et al., 2007; Li and Kirouac, 2008; Vertes and Hoover, 2008; Dong et al., 2017). Dense fiber with hM4Di/mCherry immunofluorescence was observed in the dorsomedial and medial aspect of the NAcSh (Fig. 5B) in the same general area where the injector cannula used for CNO administrations was localized (Fig. 5C).

**Fig. 6:**
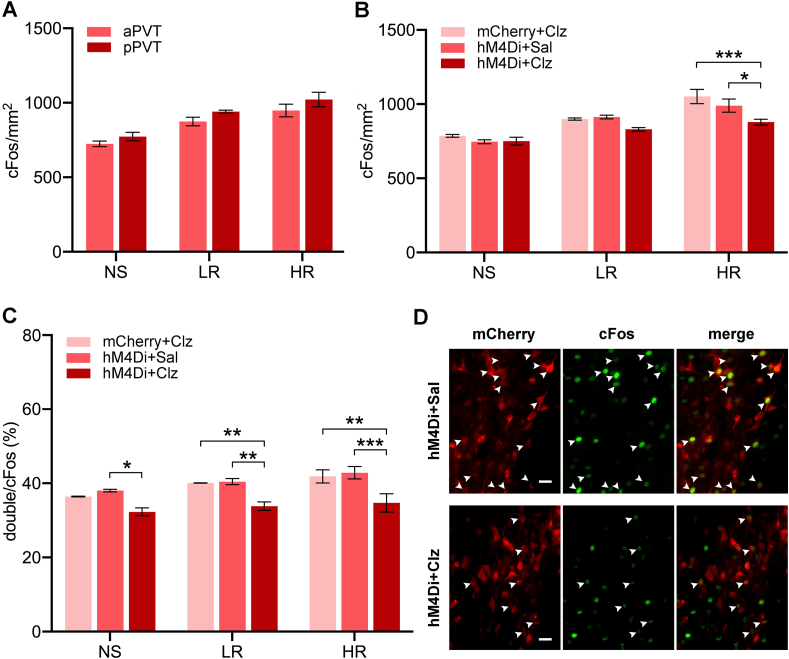
Number of PVT neurons immunolabeled for cFos following the social approach-avoidance test. (A) The number of cFos positive nuclei per mm^2^ in the anterior (aPVT) and posterior PVT (pPVT) in saline treated rats expressing hM4Di. (B) Number of cFos positive nuclei per mm^2^ in the PVT. (C) Percentages of PVT neurons double-labeled for cFos and mCherry. (D) Representative images of the PVT showing mCherry + neurons, cFos + neurons, and double-labeled cFos+/mCherry + neurons in an hM4Di + Sal and an hM4Di + Clz case. Data are means ± SEM. *p < 0.05, **p < 0.01, ***p < 0.001. Group size *n* = 4 cases for each bar. Scale bar, 50 μm.

It does not appear that the aPVT and pPVT were differentially activated by the SAA test as evidence by the lack of differences in cFos expression between these two regions in rats expressing hM4Di treated with saline (Fig. 6A, aPVT vs pPVT in NS rats, *p* = 0.66; in LR rats, *p* = 0.42; in HR rats, *p* = 0.32). Analysis of the number of cFos expressing nuclei in the PVT following the SAA test was used to assess if the PVT was preferentially activated in the HR group and whether chemogentic inhibition reduced activity of PVT neurons. There were main effects for shock groups for the total number of cFos nuclei in the PVT (Fig. 6B, *F*_(2,27)_ = 50.88, *p* < 0.001) and chemogenetic inhibition (*F*_(2,27)_ = 10.02, *p* < 0.001). The number of cFos nuclei was reduced in the HR + hM4Di group treated with clozapine compared to the same group treated with saline (*p* = 0.005) and to the HR + mCherry group treated with clozapine (*p* < 0.001). The number of neurons expressing the cFos protein and hM4Di/mCherry in the PVT following the last SAA test was also quantified to further validate the intersectional strategy (Fig. 6C and D). There were main effects for shock groups (*F*_(2,27)_ = 7.86, *p* = 0.002) and chemogenetic inhibition (*F*_(2,27)_ = 23.63, *p* < 0.001). Similar to the number of cFos nuclei, the proportion of double-labeled hM4Di/mCherry and cFos neurons was reduced in the HR + hM4Di group treated with clozapine compared to the same group treated with saline (*p* < 0.001) and to the HR + mCherry group (*p* < 0.001).

### 3.4 Evidence that PVT input to the dynorphin-expressing neurons in the NAcSh mediates stress-induced social avoidance

**Fig. 7:**
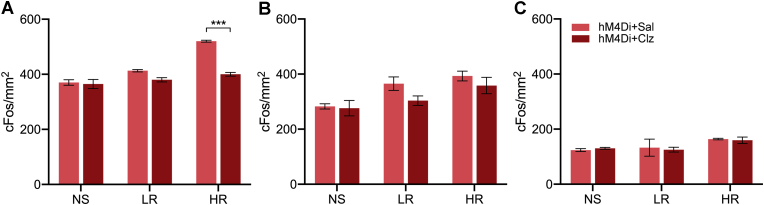
Number of cFos nuclei in areas innervated by the PVT following the social approach-avoidance test. Regions quantified are the NAcSh (A), BSTDL (B), and CeL (C). Data are means ± SEM. ***p < 0.001. Group size *n* = 4 cases for NAcSh and BSTDL, *n* = 3 to 4 cases for CeL.

The number of cFos positive nuclei in selective stereotaxic levels of the NAcSh, BSTDL and CeL are shown in Fig. 7. There was a significant difference between shocked groups in the NAcSh (Fig. 7A, *F*_(2,18)_ = 54.16, *p* < 0.001) with the HR group having more cFos than the NS and LR groups (*p* < 0.001). There was also an effect for the treatment conditions (*F*_(1,18)_ = 50.37, *p* < 0.001) and an interaction between the treatment condition and the shock groups (*F*_(2,18)_ = 21.67, *p* < 0.001). The number of cFos nuclei was reduced in the HR + hM4Di group treated with clozapine compared to the saline treated group (*p* < 0.001). For the amount of cFos in the BSTDL, there was a significant difference between shock groups (Fig. 7B, *F*_(2,18)_ = 9.42, *p* = 0.002) with the HR group having more cFos than NS group (*p* = 0.001). No significant effect was found for chemogenetic treatments in the number of cFos nuclei in BSTDL (*F*_(1,18)_ = 3.60, *p* = 0.07). Groups with shock treatment had more cFos nuclei in the CeL (Fig. 7C, *F*_(2,14)_ = 4.13, *p* = 0.039) while no statistical difference was observed between different shock groups (*post-hoc* tests between NS, LR, and HR, all *p* > 0.05). No significant effect was found in the number of cFos nuclei in the CeL between different chemogenetic treatment groups (*F*_(1,14)_ = 0.03, *p* = 0.87).

**Fig. 8:**
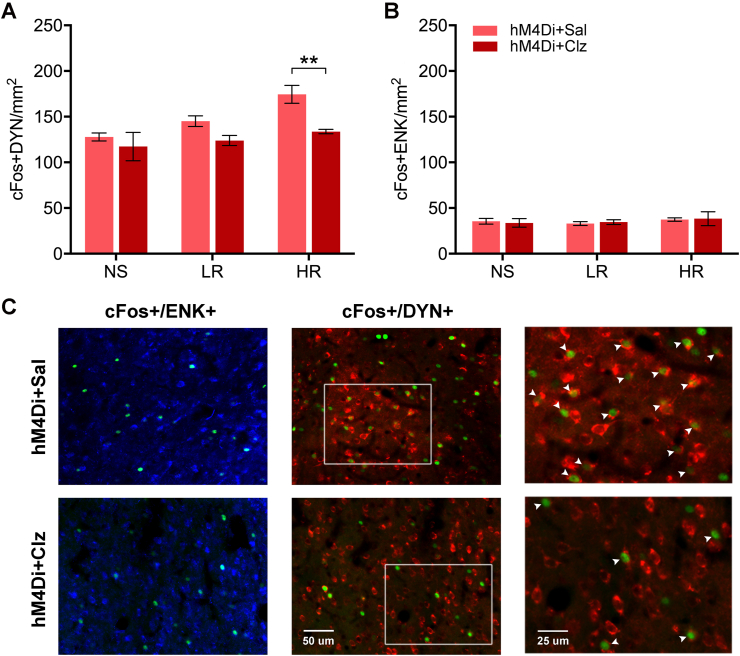
Number of double-labeled cFos neurons in the NAcSh expressing opioid neuropeptides following the social approach-avoidance test. (A) For dynorphin (DYN), the number of cFos+/DYN + double-labeled NAcSh neurons per mm^2^. (B) For enkephalin (ENK), the number of cFos+/ENK + double-labeled NAcSh neurons per mm^2^. (C) Representative images of the NAcSh showing cFos+ (green) and ENK+ (blue), and DYN+ (red) neurons in an hM4Di + Sal and an hM4Di + Clz case. Data are means ± SEM. **p < 0.01. Group size *n* = 4 cases for each bar. Scale bars, 25 and 50 μm. (For interpretation of the references to color in this figure legend, the reader is referred to the Web version of this article.)

The number of neurons that were labeled for cFos with either enkephalin or dynorphin in the NAcSh was also quantified to identify the potential involvement of the two major subtypes of medium spiny neurons in stress-induced social avoidance. There were main effects for shock groups for the number of cFos positive nuclei and dynorphin neurons (Fig. 8A, *F*_(2,18)_ = 7.07, *p* = 0.005) and chemogenetic inhibition (*F*_(1,18)_ = 12.28, *p* = 0.003) with the HR + hM4Di group treated with clozapine clearly having fewer double-labeled neurons than the same group treated with saline (*p* = 0.009). No statistical difference was found in NS or LR groups with different chemogenetic treatments. The number of cFos and enkephalin-labeled neurons was not statistically different across the different shock conditions (Fig. 8B, *F*_(2,18)_ = 0.53, *p* = 0.60) or chemogenetic conditions (*F*_(1,18)_ = 0.005, *p* = 0.95).

## 4 Discussion

Anxiety is regulated by brain circuits engaged in hierarchical control of defensive strategies in which a cortical network evaluates potential risks and initiates defensive responses via projections to subcortical regions (Kim et al., 2013; Adhikari, 2014; Duvarci and Pare, 2014; Calhoon and Tye, 2015; Fox et al., 2015; Fox and Shackman, 2017). Exposure of rodents to a single episode of footshock stress can have lasting effects on behavior including enhanced social avoidance (Louvart et al., 2005; Siegmund and Wotjak, 2007; Mikics et al., 2008; Chen et al., 2012). The PVT is activated by stress (Hsu et al., 2014; Kirouac, 2015) and has been shown to contribute to the behavioral responses indicative of a heightened anxiety state following footshock stress (Li et al., 2010a; Dong et al., 2015). The present investigation demonstrates that chemogenetic inhibition of PVT neurons that project to the NAcSh attenuates the enhanced social avoidance displayed by rats exposed to footshocks. The contribution of a PVT-NAcSh projection to avoidance appears to be uniquely related to social threats since the behavioral responses in the open field and shock context were unaffected by the same treatment. These results indicate that the PVT specifically enhances the saliency of the social threats and/or social avoidance following the exposure of rats to moderately intense footshocks.

### 4.1 Methodological considerations

There are multiple advantages of the intersectional chemogenetic approach used in the present study. First, hM4Di was transduced specifically in PVT neurons and not other midline thalamic nuclei located immediately adjacent to the PVT. It is difficult to target the PVT without affecting other thalamic nuclei using a non-intersectional viral injection or with injections of pharmacologically active substances. The specificity of the intersectional viral injection method is clearly evident by the lack of transduced neurons in thalamic nuclei outside the PVT including the paratenial nucleus which projects to the core of the accumbens (Vertes and Hoover, 2008). Another advantage is that hM4Di was transduced in PVT neurons that project robustly to the NAcSh. This is especially important since many of the neurons in the PVT that project to the NAcSh also send collaterals to the BSTDL and CeL (Dong et al., 2017). Injections of AAV8-hM4Di were done in both the anterior and posterior regions of the PVT to express hM4Di through the entire length of the PVT since neurons that project to the NAcSh originate in anterior-posterior extent of the PVT (Dong et al., 2017). It is still possible that neurons in the aPVT and pPVT may differentially control some of the behaviors examined in the present study (Hsu et al., 2014; Kirouac, 2015). However, differences in cFos expression between the aPVT and pPVT to the SAA test were not observed indicating that this is unlikely.

The ligand of choice for designer receptors like hM4Di has been CNO (Roth, 2016). However, systemic administered CNO can be converted to clozapine which then acts as the agonist that provides much of the functional activation of the designer receptor (Gomez et al., 2017). Accordingly, a low dose of clozapine (0.01 mg/kg, i.p.) was used to activate the hM4Di because this dose of clozapine acts directly on hM4Di without having sedative effects (Gomez et al., 2017). Groups of rats treated with the vehicle were included to control for the expression of hM4Di as well as the pharmacological effects of clozapine which has some affinity to serotonin and dopamine receptors (Gomez et al., 2017). Changes in cFos expression was also used to provide evidence that clozapine specifically inhibited PVT neurons expressing hM4Di. The agonist CNO was used for the local injection in the NAcSh since effective dose and volume for the NAcSh had been previously reported (Alexander et al., 2009; Zhu et al., 2016; Jaramillo et al., 2018).

### 4.2 Contribution of the PVT to fear and anxiety

The strong anatomical connections between the PVT and the BSTDL and CeL are suggestive of a role for the PVT in anxiety and fear (Li and Kirouac, 2008; Kirouac, 2015). Experimental evidence has been provided that the PVT regulates conditioned fear (Padilla-Coreano et al., 2012; Li et al., 2014; Do-Monte et al., 2015; Penzo et al., 2015) via a projection to the CeL (Do-Monte et al., 2015; Penzo et al., 2015). There is also pharmacological evidence that the PVT regulates anxiety (Li et al., 2009; Li et al., 2010a, b; Heydendael et al., 2011; Barson and Leibowitz, 2015) including the anxiety that develops following exposure of rats to footshock stress (Li et al., 2010a; Dong et al., 2015). However, the projection by which the PVT mediates stress-induced anxiety had not been examined using projection specific manipulations prior to this investigation. Our choice to investigate the contribution of the PVT neurons and fibers that project to the NAcSh was based on the finding that most neurons in the PVT project to the NAcSh (Dong et al., 2017) and that this projection system is involved in the selection of behavioral responses to motivational conflicts (Choi and McNally, 2017; Do-Monte et al., 2017; Choi et al., 2019). Fear and anxiety are similar types of emotions that can be inferred in experimental animals from the expression of stereotypical defensive behaviors (LeDoux, 2015; LeDoux and Pine, 2016). Fear is triggered by the presence of impending threats and is often experimentally defined in rodents as behavioral freezing to conditioned cues or contexts. In contrast, anxiety is a response to potential threats and is defined as avoidance of risks in situation with a motivational conflict involving both approach and avoidance tendencies (Steimer, 2002; LeDoux, 2015). In the present investigation, we found that inhibition of the PVT projection to the NAcSh attenuated the enhanced avoidance to a novel conspecific displayed by HRs while not affecting the avoidance or freezing in the open field. Chemogenetic inhibition of the PVT-NAcSh projection also had no effect on contextual fear providing further support for the conclusion that this projection regulates stress-induced social avoidance independently of other defensive behaviors.

### 4.3 Potential mechanisms for PVT mediated stress-induced social avoidance

The PVT is an area of the brain consistently identified as being activated during states of arousal and by emotionally salient cues (Kirouac, 2015; Millan et al., 2017). Single unit and calcium signal recording of neurons in the PVT during behaviorally relevant events indicate that PVT neurons may code primarily the saliency and not the valence of cues that predict aversive and appetitive outcomes (Choi and McNally, 2017; Zhu et al., 2018). In the present investigation, we found an increase in cFos expression in the PVT of HRs following the SAA test. The SAA involves a conflict situation where a novel rat presents potential social benefits and threats (Toth and Neumann, 2013). Accordingly, one possible interpretation of the findings presented here is that the footshock experience leads to changes in the PVT that make neurons in this region of the thalamus more responsive to the threat saliency of a novel rat. Our group has provided pharmacological evidence that the orexin peptides promote footshock-induced anxiety by acting at the PVT (Li et al., 2010a; Dong et al., 2015). The effect of footshock stress on orexin neurons was shown to last for weeks in susceptible rats (Chen et al., 2014). Other evidence indicates that the action of orexins on PVT neurons in response to stressful event may lead to neuroplasticity changes that may make the PVT more sensitive to novel challenges (Heydendael et al., 2011). The stress effects observed here may generalize to a variety of stressors since the latter study involved repeated exposures to swim stress and not a single episode of footshocks. We postulate that stress may make orexin neurons more responsive to arousing stimuli and enhance the sensitivity of the PVT neurons to novelty and potential threats. Accordingly, one potential mechanim for mediating an enhanced social threat saliency in HRs could involve an orexin-mediated amplification of the signals from prefrontal areas that signal social threats (Minami et al., 2017; Huang et al., 2020). Interestingly, orexins may heighten the saliency of reward cues by enhancing cue-related signals from the prefrontal cortext to the PVT (Otis et al., 2017, 2019).

Dopamine release in the nucleus accumbens can bidirectionally modulate the ability of socially salient stimuli to promote approach or avoidance of a conspecific while having no effect on non-social stimuli (Gunaydin et al., 2014). Therefore, another potential mechanim by which exposure to footshock leads to a PVT-mediated social avoidance may involve stress-induced changes in the ability of the PVT to modulate dopamine release in the NAcSh. In response to aversive condition, medium spiny neurons in the NAcSh can decrease dopamine release in this region of the striatum via a dynorphin-mediated mechanism (Newton et al., 2002; Barrot et al., 2005; Nestler and Carlezon, 2006; Bruchas et al., 2008; Land et al., 2008; Al-Hasani et al., 2015; Castro and Bruchas, 2019; Margolis and Karkhanis, 2019; Tejeda and Bonci, 2019). In the present study, we found that there was an increase in the number of dynorphin neurons in the NAcSh activated in the HR group exposed to the SAA test. More importantly, this number was significantly attenuated in rats expressing hM4Di that were treated with clozapine. This latter finding supports our conclusion that chemogenetic inhibition of the PVT-NAcSh projection attenuates social avoidance by inhibiting a PVT-mediated activation of dynorphin neurons. Accordingly, the PVT could promote social avoidance by activating dynorphin neurons that modulate the release of dopamine in the NAcSh. More direct evidence to support this hypothesis could be provided using cell-specific manipulations that permit both loss and gain of function of these neurons in footshock stressed rats exposed to a SAA test. This could also be investigated using pharmacological agent that block kappa opioid receptor closely associated with dynorphin function (Bodnar, 2020).

The BSTDL and CeL have been shown to be involved in fear and anxiety (Walker et al., 2003, 2009; Kalin et al., 2004; Cai et al., 2012; Jennings et al., 2013; Kim et al., 2013; Ventura-Silva et al., 2013; Pleil et al., 2015; Ahrens et al., 2018; Asok et al., 2018; Normandeau et al., 2018). The present paper provides chemogenetic evidence that PVT neurons that project to the NAcSh mediate social avoidance but not the other behavioral changes. An unresolved question is whether the anxiety-like behaviors in the open field and the strong contextual fear produced by footshocks are mediated by neurons in the PVT that project to the BSTDL or the CeL or fiber collateral to the BSTDL and CeL that originate from the same PVT-NAcSh projection neurons that mediate social avoidance. A lack of an effect on contextual fear could be considered surprising considering the well-characterized role of a PVT projection to CeL in conditioned fear to auditory cues (Do-Monte et al., 2015; Penzo et al., 2015). The negative findings regarding contextual fear in the present study may simply reflect the fact that fear conditioning to discrete cues may involve different components of the fear circuits. Further studies that involve inactivation of PVT neurons that project to the BSTDL and CeL or the collaterals to these areas will be necessary to clearly establish if the PVT projections to the BSTDL and CeL have a role in contextual fear or the stress-induced changes in open field behavior. A hypothesis that the same PVT neuron contributes to a situation-specific social avoidance and other stress-induced behaviors via collaterals to different subcortical regions is intriguing considering the presence of divergent projections to the NAcSh, BSTDL and CeL (Dong et al., 2017).

## Conflicts of interest

The authors declare no competing financial interests.

## CRediT authorship contribution statement

**Xinwen Dong:** Investigation, Methodology, Draft Preparation. **Sa Li:** Investigation, Draft Preparation. **Gilbert J. Kirouac:** Conceptualization, Methodology, Draft Preparation.
